# Clinical Notes

**Published:** 1873-04-15

**Authors:** F. K. Bailey

**Affiliations:** Knoxville, Tenn.


### Clinical Lectures.

### CLINICAL NOTES.

BY F. K. BAILEY, M.D., KNOXVILLE, TENN.

Prefatory to some remarks upon cases which have come under my eye during the winter just closing, I will state that the season has been almost unprecedentedly cold and rainy. The traditional sunny days in January did not occur, and the plow in consequence was idle. The terrible storm-blasts, which have rendered the northern belt of our country so inclement, touched this region, even though protected more or less by the Cumberland mountains. In due course of its march the epizootic reached us, sickening the horses and bringing the riders to their feet. The disease was of a mild character, and appeared reluctant to come at all, and prevailed on every side before reaching this immediate locality. There are some neighborhoods in this and adjoining counties that are still untouched.

During its prevalence and its subsidence an epidemic catarrhal affection seized the whole community, affecting every family and every individual.

The attack was characterized by a sudden stoppage of nasal and bronchial secretion, with considerable febrile reaction, constipation, and red, scanty urinary secretion. Soon, the mucous surfaces began to secrete freely, but without much abatement of unpleasant symptoms.

A brisk cathartic, followed with diuretics, would generally relieve inordinate symptoms; but sometimes bronchitis and broncho-pneumonia set in, with the usual concomitants.

During the last autumn there was more than the usual tendency to febrile affections, and an occasional case of an attempt at the intermittent type gave variety to our practice.

Small-pox has invaded our locality. The first case was in the person of a clergyman, who was taken after returning from Memphis, where he was exposed.

He recovered, but from some unfortunate circumstances his two youngest children took the disease and died. The eldest, a girl of nine, lived but a short time after the eruption appeared; and the other, about a year old, died from the shock—and possibly it might not have had small-pox. An adult member of the family had varioloid; but the disease went no further. A railroad man came home with the disease, and died ; but no other person in the family took it. A negro man who had washed some of the clothes of this man took the disease and died.

This is the extent of the visitation; and the remark made a year or more ago by Dr. Frank A. Ramsey, that small-pox could not thrive on Knoxville soil, seems verified.

There are great numbers of unvaccinated yet going about; and nothing but a marvelous exception in our favor can keep it from invading the city to a terrible extent.

March 16.—Small-pox has again appeared, but, with one exception, confined to the negroes.

RHEUMATISM.

This is a disease so common that a description is hardly necessary.

Although so many persons complain of rheumatic symptoms—and we daily meet with individuals laboring under one form or other of the disease—still, a well marked case of arthritic rheumatism, with characteristic febrile attendants and constitutional phenomena, is not so often encountered.

November 21, 1872, I was called to visit a lady about thirty-two, a native of Tennessee, married more than five years, but has never been pregnant. Found her with fever of a medium grade; pulse 108; tongue coated at the base with a yellow fur, but clean and reddish at the tip; countenance showing an anxious appearance; and the face of a bronzed hue, although the temperament and complexion do not indicate "biliousness" as peculiarly marked.

The alimentary canal is in a torpid condition, which she informs me has been obvious for some weeks.

The stools are scanty and scybalous, only passing off at irregular intervals. The urine scanty, high-colored, and causing some irritation in being voided. Complains of pain and a sense of weight in the lumbar region. Besides the general morbid manifestations above described, there is swelling of the right knee. Above the joint anteriorly there is puffiness to a considerable extent. Upon the inside of the joint, and around the patella, not only hard swelling, but excessive tenderness to the touch; and so extremely sensitive is the whole arthritic tissue that motion is out of the question. Even at complete rest there is severe pain through the whole joint, extending downwards along the leg. As would be expected, there was want of sleep at night, and a general restlessness at all hours.

Prescribed Dover's powders to be taken at night; also two comp. rhubarb pills, and the same next morning. The following mixture was directed to be taken in teaspoonful doses four times daily:

℞.—Iod. potassii,

Bi-carb. potassa, } aa. ʒ i.

Syr. senegac, ℥ i.

Tinct. actea cimicifugæ, ℥ ss.

The affected joint to be kept warmly covered and bathed with a stimulating liniment of aquæ ammonia and olive oil.

November 22d.—Found the pills had moved the bowels, but no other change to be noted. To continue treatment suggested yesterday, except to suspend Dover's powders, and substitute as follows:

℞.—Bromide potassii, ʒ ii.

Syr. zinziber, ℥ ii.

Teaspoonful every four hours, commencing at noon.

November 24th.—Some improvement. Has rested a little during the last two nights. Bowels open, urine still scanty. To continue same remedies.

November 26th.—Much less pain in the knee, but swelling not diminished. Bowels not open. Some fever every day after 3 P.M., lasting till past midnight. Thirst not marked, but a desire for cool drinks. Tongue cleaning somewhat. Pulse about 100, soft, but quick. Respiration not hurried, nor are there any unpleasant chest symptoms to indicate cardiac complications present or imminent. To take rhubarb pills as before, and continue both mixtures.

November 30th.—Although daily visiting my patient, and carefully watching symptoms, there has been but little material change. The evening exacerbations have been regular in their recurrence, with a slight moisture about the body towards morning. She has been able to sleep some at night since taking the bromide. The bowels have been opened, as occasion required, by the pills. At times has complained of pain in the iliac region, and informs me that she has a somewhat free leucorrhœal discharge, which, by the way, appears to have been a source of trouble to her for years. The soreness about the knee principally confined to the inside, with a sore spot about two inches in extent which will not tolerate the slightest touch. To suspend the iod. potassii mixture, and continue the bromide.

December 3d.—Since last report there has been a decided improvement in the knee. There is less swelling, and the patella can be traced for the first time. The appetite, which for some time was indifferent, is somewhat improving. Urine quite free and more copious, as the patient says, than it has been for weeks. Tongue cleaning, and the bronzed hue to the face entirely gone.

For some days past there has been a slight sense of chilliness at the time that the fever had commenced, with more of the night-sweating. Prescribed as follows:

℞.—Sulph. quinia, ℈j.

Iod. potassii, ℈ij.

Tinct. cinch. comp., ℥ iss.

Mix. Give teaspoonful every four hours.

December 5th.—Very much improved. The knee, although still swollen, is susceptible of motion and handling. Can sit up in the bed and move about without causing pain. Appetite very good. Bowels move every day of their own accord, and the urine is still quite copious and of natural color.

Leucorrhœal discharge still continues. To continue quinine mixture, and to move the affected limb as much as possible without causing pain.

December 7th. — Still better; but little fever at night; bowels open, and appetite still good. Tongue clean; the countenance has assumed a natural appearance, having none of the anxious cast at first observed. To continue mixture.

December 9th.—No fever at all last night, and but slight sweating. Pulse natural. Swelling nearly left the knee, except the puffiness at the upper portion, and some at the point where the soreness had been so great. Continue mixture.

December 12th.—Still improving.

March 1st, 1873.—Saw the woman, who informs me that she has wholly recovered her health, and feels not the slightest lameness in the affected joint.

